# Contrast-enhanced ultrasound for reclassification of moderate-risk carotid plaques (Plaque-RADS 3)

**DOI:** 10.1186/s12880-026-02564-0

**Published:** 2026-07-13

**Authors:** Ruifeng Liu, Jiachun Xie, Zhenli Huang, Liya Wei

**Affiliations:** 1 Department of Ultrasound, Peking University Shenzhen Hospital, No. 1120 Lianhua Road, Futian District, Shenzhen, Guangdong Province 518036 China; 2 Macao Medical Center of Peking Union Medical College Hospital, Macao, China

**Keywords:** Plaque-RADS, Contrast-enhanced ultrasound, Symptomatic carotid plaques

## Abstract

**Background:**

The Carotid Plaque-RADS system has been proposed to standardize carotid plaque evaluation recently. However, the carotid plaques classified into plaque-RADS 3 share considerable heterogeneity, which poses challenges for clinical management. This research aimed to investigate the supplementary value of contrast-enhanced ultrasound (CEUS) in assessing these moderate-risk carotid plaques designated as plaque-RADS 3.

**Methods:**

A retrospective diagnostic analysis was conducted on 194 patients (270 plaques) who underwent carotid plaque CEUS from January 2020 to May 2024. Plaque features and patients’ characteristics were retrieved. CEUS videos were independently reviewed by two senior radiologists, and intraplaque neovascularization (IPN) intensity was graded semi-quantitatively using a four point score system (0 = no enhancement; 1 = spotty enhancement; 2 = spotty and 1–2 short linear enhancements; 3 = linear enhancement traversing the plaque). Plaques were subclassified into 3a, 3b, and 3c groups based on the Carotid Plaque-RADS criteria proposed by saba et al. Differences in IPN among subgroups and their association with symptomatic plaques were analyzed using multivariate logistic regression.

**Results:**

Among 270 plaques, 92 were classified as 3a, 146 as 3b, and 32 as 3c. No significant differences were observed in age, gender, plaque echogenicity, length, thickness, or lumen stenosis severity among groups. Multivariate logistic regression identified luminal stenosis (OR = 2.129, *p* < 0.001), plaque thickness (OR = 2.429, *p* < 0.001), length (OR = 1.033, *p* < 0.001), and IPN (OR = 1.884, *p* = 0.012) as independent predictors of symptomatic plaques. IPN grading differed significantly across groups (*p* < 0.001), with 3a plaques showing a higher proportion of low (0–1) IPN scores (40.2% vs. 7.5% in 3b and 6.3% in 3c, *p* < 0.001). Intra- and inter-observer agreement for IPN grading was good (kappa = 0.762 and 0.704, respectively).

**Conclusions:**

Approximately 40.2% of 3a plaques exhibited low IPN score, correlating with possibly reduced symptomatic risk. Despite all Plaque-RADS 3 entities described as stroke-moderate-risk, CEUS-assessed IPN enables further risk stratification-A subset of 3a plaques may warrant reclassification to lower-risk categories.

**Clinical trial number:**

Not applicable.

## Introduction

Stroke, a severe cardiovascular disease associated with high mortality and disability, mainly composed of ischemic type and hemorrhagic type. Ischemic stroke (IS), accounting for 80%-85% of all strokes, are frequently attributed to carotid artery atherosclerosis, especially unstable plaques [1, 2]. These vulnerable plaques are prone to rupture, leading to arterial embolization, and are characterized by defined features including inflammatory cell infiltration, thin fibrous cap overlying large lipid-rich necrotic cores (LRNC), intraplaque fissures, surface thrombus formation, and intraplaque hemorrhage (IPH) [3]. Accurate assessment of plaque characteristics to determine future stroke risk remains a clinical conundrum [4]. Recently, the Carotid Plaque-RADS (Reporting and Data System) has been proposed to standardize carotid plaque evaluation, enhancing inter-institutional reporting homogeneity and facilitating data exchange. This scoring system, applicable to ultrasound, MRI, and CT imaging, classifies individual plaques into four categories (Carotid Plaque-RADS 1 [no plaque] to Carotid Plaque-RADS 4 [plaques with complex features]) based on thickness, composition, and morphology. This classification of plaques could directly indicate the risk of stroke. A report stating “Plaque-RADS 4” is immediately understood to represent a high-risk, vulnerable plaque, signaling a need for intensified treatment. Its limitations, including operator dependency, the need for further validation, and modality-specific constraints, mean that it should be used as a complementary tool, not a replacement for clinical judgment. Researches to broaden the new tool’s application in different clinical scenes are in development [5, 6]. Notably, Plaque-RADS category 3 plaques are specifically focused in our study due to their heterogeneous nature and uncertain clinical management, whereas categories 2 and 4 exhibit more established risk profiles and are less likely to benefit from additional stratification in the present context [7].

The Plaque-RADS framework emphasizes the importance of ancillary features (AnFe), which enable subclassification of individual plaques. Among these features, intraplaque neovascularization (IPN) has been extensively validated as a marker of plaque instability, and in the meantime, contrast-enhanced ultrasound (CEUS) demonstrates high efficacy in visualizing neovascularization. Recent systematic reviews have underscored the role of contrast-enhanced ultrasound (CEUS) in detecting IPN and its correlation with histologically confirmed plaque vulnerability [8, 9]. A recent commentary has further emphasized the potential of CEUS-assessed IPN in refining plaque vulnerability assessment within the Plaque-RADS framework [10]. Therefore, this study aims to evaluate the supplemental diagnostic value of CEUS in refining risk stratification and reclassification of Carotid Plaque-RADS Category 3 plaques.

## Methods and materials

### Study population

**Fig. 1:**
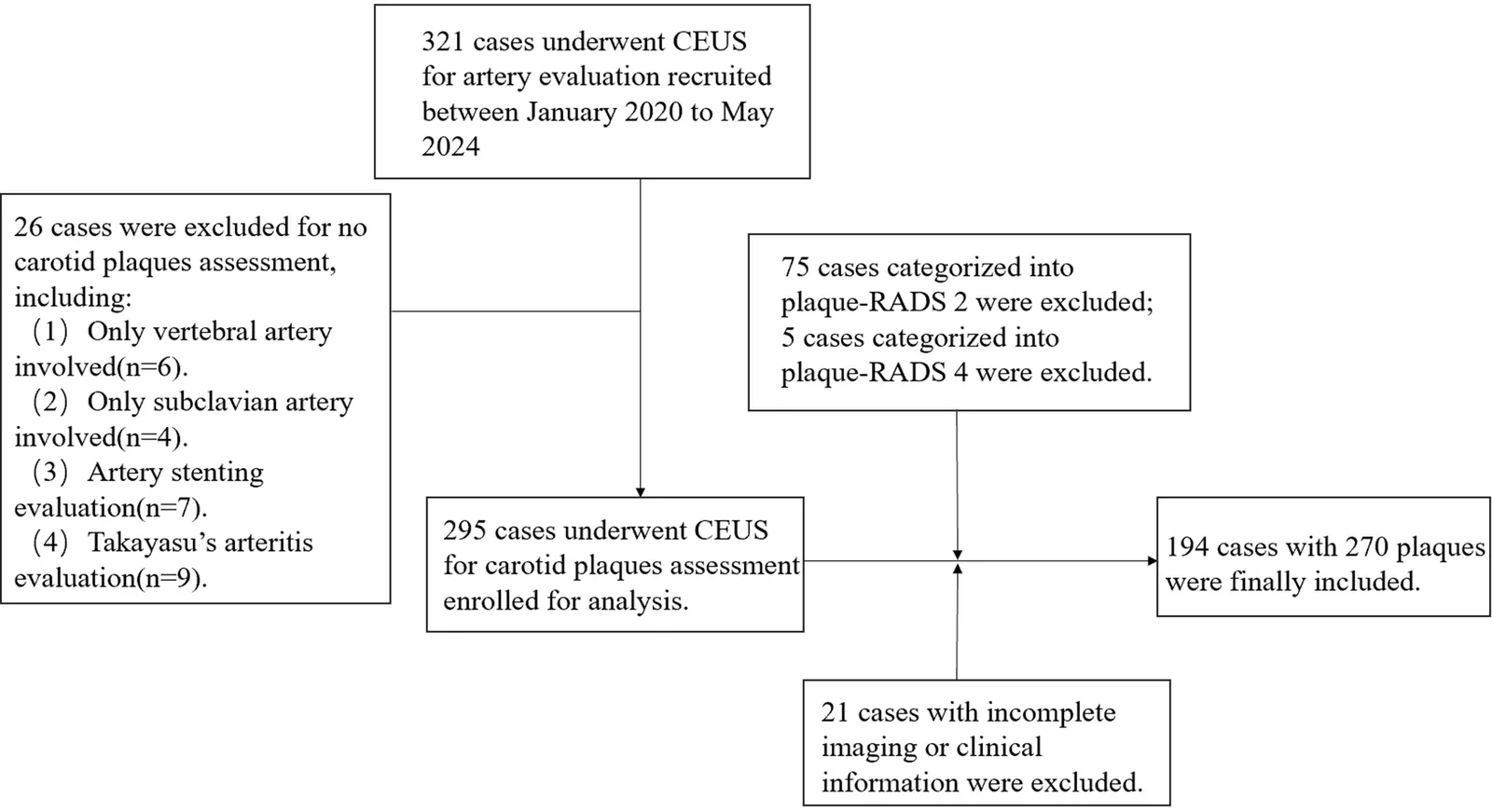
Flow scheme of patients’ selection

This retrospective cohort study evaluated 321 patients who underwent carotid plaque contrast-enhanced ultrasound (CEUS) at Peking University Shenzhen Hospital between January 2020 and May 2024. Inclusion criteria were: (1) age ≥ 18 years; (2) presence of Plaque-RADS Category 3 carotid plaques confirmed by two-dimensional (2D) ultrasound; and (3) no prior surgical intervention; (4) complete clinical data. Exclusion criteria included: (1) cases exclusively involving vertebral/subclavian artery plaques or post-carotid stent implantation; (2) severe hepatic/renal dysfunction or contrast agent allergy; and (3) poor image quality (e.g., acoustic shadowing from calcified plaques interfering with CEUS visualization). Ultimately, 194 patients (270 plaques) met the inclusion criteria (Fig. 1). As an exploratory retrospective analysis, no formal sample size calculation was performed a priori. The study aimed to include all eligible patients with high-quality CEUS imaging during the study period to maximize available data. Patients’ plaques were classified as symptomatic or asymptomatic according to the onset of transient ischemic attack (TIA) symptoms or imaging-confirmed non-thrombotic ischemic stroke (IS) within the previous 8 weeks. The study was approved by the Ethics Committee of Peking University Shenzhen Hospital (Approval No. 2024 − 109), with waived informed consent.

### 2D ultrasound and plaque-RADS classification

Ultrasound examinations were performed using a Philips EPIQ 7c system equipped with an L9-3 linear transducer (Philips Medical Solutions), adhering to the *Guidelines for Carotid Plaque Assessment* by the American Society of Echocardiography. Protocol details: (1) Patients were positioned supine with the head turned contralateral to the target plaque for optimal neck exposure; (2) 2D ultrasound measured maximum wall thickness (MWT), plaque length, and luminal stenosis (calculated via North American Symptomatic Carotid Endarterectomy Trial [NASCET] criteria); (3) Plaque echogenicity was categorized as hyperechoic, isoechoic, or hypoechoic based on the predominant component (> 50% of the plaque area). Plaques with equivocal or balanced mixed echogenicity (no component > 50%) were excluded to ensure reliable classification. Plaques were classified as *Carotid Plaque-RADS*: Category 3a: Defined by a thick, intact fibrous cap (FC), appearing as a hyperechoic cap-like structure on the plaque surface, and a moderate to large hypoechoic or anechoic area within the plaque probably corresponding to lipid-rich necrotic core (LRNC); Category 3b: Characterized by a thin or indistinct fibrous cap, visualized as a juxtaluminal black area (JBA), on the plaque surface, and a moderate to large hypoechoic or anechoic area within the plaque probably corresponding to lipid-rich necrotic core (LRNC); Category 3c: Ulcerated plaques with surface irregularities, regardless of plaque thickness, and in the absence of IPH, FC disruption, or intraluminal thrombus.

### CEUS protocol and consistency evaluation

CEUS was performed using SonoVue^®^, with 1.2 mL bolus via antecubital vein and continuous imaging for ≥ 120 s. For bilateral carotid lesions, a 10-minute interval was maintained between injections. Imaging parameters included a mechanical index (MI) of 0.08–0.12, frame rate of 15 Hz, and focal zone positioned at plaque level. Intraplaque neovascularization was semi-quantitatively graded (0–3) referring to a previously reported system [11]: 0-No enhancement, 1-Focal enhancement confined to the adventitial side, 2-Focal enhancement at the plaque shoulder and/or intima side, 3- Enhancement involving the plaque core or extensive intraplaque enhancement. The semi-quantitative IPN grading system, albeit in different forms, has been widely validated against histologic microvessel density in previous studies [12–14]. Due to the retrospective nature of this work and the prevailing shift toward stent-based interventions, direct histopathologic correlation was not feasible in the cohort. CEUS video analysis was performed by two radiologists, each with over 5 years of experience in vascular ultrasound, who were blinded to all clinical data. Dynamic CEUS clips were reviewed in random order via the PACS system. Intra- and inter-observer agreement was assessed. For intra-observer consistency, all images were re-evaluated by the same observer 30 days post-initial assessment. Inter-observer agreement was determined by two independent observers using identical dynamic images and scoring criteria.

### Statistical analysis

Data were analyzed using SPSS 25.0. Continuous variables are summarized as mean ± standard deviation and median ± interquartile range according to normality verifed by Shapiro–Wilk test. Mann–Whitney U test was used for comparison of biochemical parameters. Categorical variables were analyzed via chi-square or Kruskal-Wallis tests and reported as frequencies and percentages. Group comparisons for continuous variables employed ANOVA (with Bonferroni post-hoc tests) or Kruskal-Wallis tests (with Dwass-Steel-Critchlow-Fligner [DSCF] post-hoc tests). Intra- and inter-observer agreement for IPN grading was evaluated using Cohen’s kappa. The κ value was interpreted according to Landis and Koch’s report [15]: poor, κ < 0.20; fair, 0.20 < κ < 0.40; moderate, 0.40 < κ < 0.60; good, 0.60 < κ < 0.80; and very good, 0.80 < κ < 1.00. Multivariate logistic regression identified independent risk factors for symptomatic plaques, with odds ratios (ORs) and 95% confidence intervals (CIs) reported. A two-tailed *p*-value < 0.05 denoted statistical significance.

## Results

### Patients characteristics and plaque features

This study included 194 patients with a total of 270 Plaque-RADS Category 3 carotid plaques: 92 (34.1%) classified as 3a, 146 (54.1%) as 3b, and 32 (11.9%) as 3c. No significant differences were observed among the three groups in baseline clinical or biochemical variables, including age, sex, smoking history, alcohol consumption, hypertension, diabetes, family history of cardiovascular disease, BMI > 24, or lipid profiles (Table 1). Similarly, ultrasound-assessed plaque characteristics—such as plaque location, echogenicity, thickness, length and degree of luminal stenosis—showed no significant intergroup differences (Table 2).

**Table 1:** Comparison of demographic and clinical parameters among patients with carotid plaques classified as plaque 3a, 3b and 3c

Group variables	3a (*n* = 92)	3b (*n* = 146)	3c (*n* = 32)	F-value	significance
Gender (male)	77(83.7%)	120(82.2%)	30(93.8%)		0.268
Age (y)	66.5 ± 9.6	65.5 ± 8.9	67.8 ± 9.5	0.879	0.416
Smoking	37(40.2%)	64(43.8%)	18(56.3%)		0.289
Alcohol intake > 10 unit/wk	41(44.6%)	67(45.9%)	12(37.5%)		0.688
Hypertension	61(66.3%)	91(62.3%)	21(65.6%)		0.808
Diabetes mellitus	27(29.7%)	42(28.8%)	12(37.5%)		0.618
Family history of cardiovascular events	20 (21.7%)	36 (24.7%)	7 (21.9%)		0.856
BMI>24 (kg/m^2^)	55(59.8%)	82(56.2%)	15(46.9%)		0.447
Triglycerides (mmol/L)	1.84 ± 1.36	1.74 ± 1.79	1.78 ± 1.18	0.110	0.896
Cholesterol (mmol/L)	4.99 ± 1.24	5.05 ± 1.22	4.77 ± 1.21	0.738	0.479
LDL (mmol/L)	3.28 ± 0.91	3.32 ± 0.80	3.18 ± 0.91	0.394	0.674
HDL (mmol/L)	1.17 ± 0.24	1.19 ± 0.27	1.15 ± 0.23	0.450	0.638
Statin treatment	51(55.4%)	73(50%)	14(43.8%)		0.483
Antihypertensive treatment	56(60.9%)	85(58.2%)	17(53.1%)		0.741
Antiplatelet/coagulants treatment	26(28.3%)	24(16.4%)	8(25%)		0.084

**Table 2:** Comparison of ultrasound parameters among carotid plaques grouped into plaque 3a, 3b and 3c

Group US variables	3a (*n* = 92)	3b (*n* = 146)	3c (*n* = 32)	F-value	*p*-value
Location(right)	45(48.9%)	68(46.6%)	18(56.3%)		0.609
Stenosis degree (< 50%/50%-69%/70%-99%)	47(51.1%)/ 19(20.7%)/ 26(28.3%)	71(48.6%)/ 28(19.2%)/ 47(32.2%)	14(43.8%)/ 6(18.8%)/ 12(37.5%)		0.904
Echogenicity (hyper/iso/hypo-echo^*^)	16(17.4%)/ 50(54.3%)/ 25(27.2%)	11(7.5%)/ 74(50.7%)/ 61(41.8%)	5(15.6%)/ 16(50.0%)/ 10(31.3%)		0.067
Thickness	4.34 ± 1.32	4.38 ± 1.31	4.69 ± 1.39	0.812	0.445
Length	29.9 ± 22.7	27.2 ± 19.7	32.0 ± 23.5	0.895	0.410
intraplaque neovascularization grading (0–1/2/3)+	37(40.2%)/ 31(33.7%)/ 24(26.1%)	11(7.5%)/ 57(39.0%)/ 78(53.4%)	2(6.3%)/ 14(43.8%)/ 16(50.0%)		<0.001
Structural anomalies on echocardiography	35(61.4%)	59(63.4%)	12(57.1%)		0.860
Tissue doppler anomalies on echocardiography	22(39.3%)	51(55.4%)	10(50%)		0.162

*: defined by the main echogenicity (>50%) of plaques

### Plaque CEUS characteristics and reliability evaluation

**Fig. 2:**
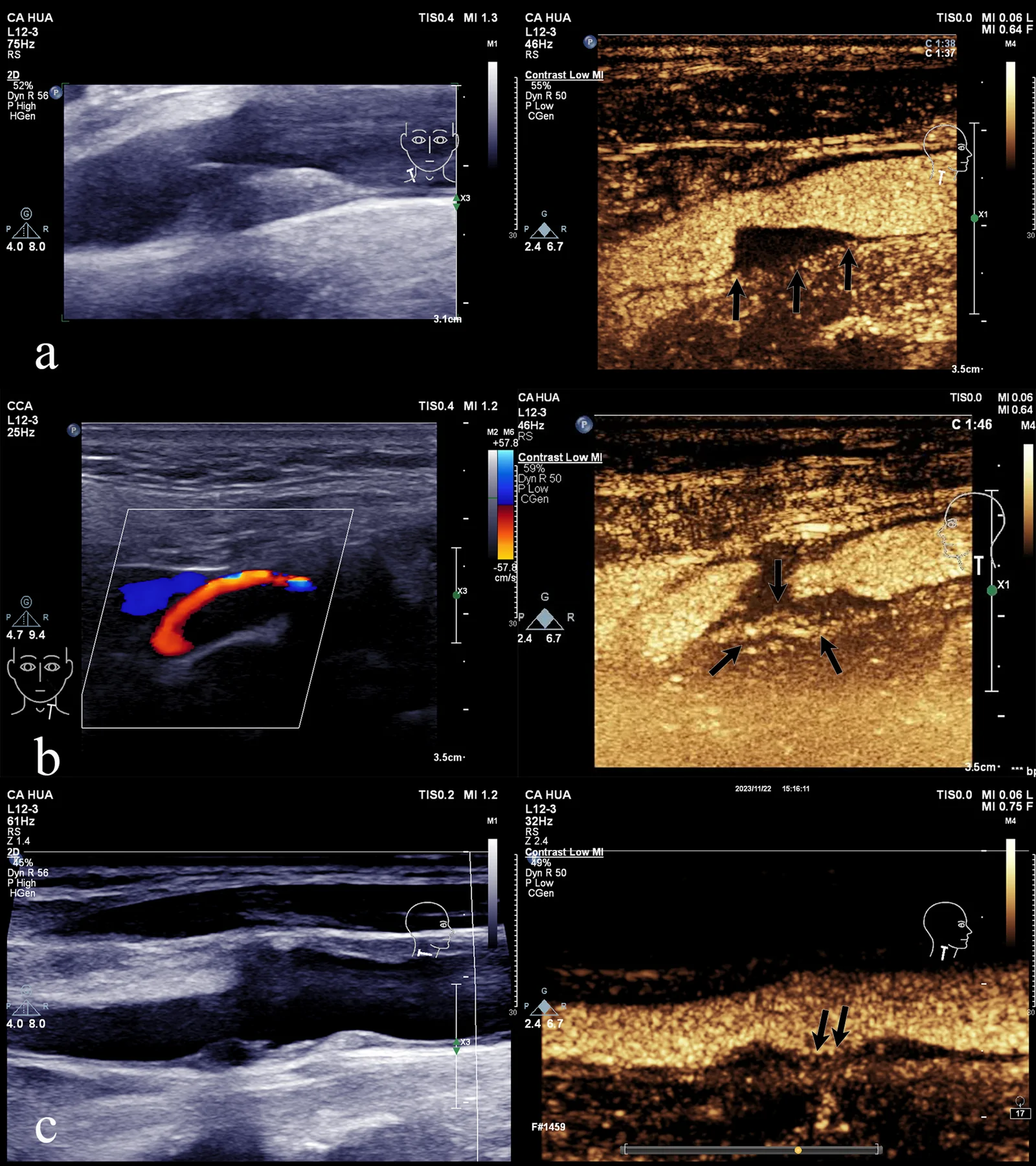
(**a**) A carotid plaque, categorized as plaque-RADS 3a, with focal enhancement confined to the adventitial side (arrows) scoring 1; (**b**) A carotid plaque, categorized as plaque-RADS 3b, with extensive enhancement (arrows) scoring 3; (**c**) A carotid plaque, categorized as plaque-RADS 3c, with focal enhancement at the shoulder and the intima side (arrows) scoring 2

Intraplaque neovascularization (IPN) grading exhibited significant disparity across groups. The proportion of plaques with low IPN scores (0–1) was significantly higher in the 3a subgroup (40.2%) compared with the 3b (7.5%) and 3c (6.3%) subgroups (*p* < 0.001) (Fig. 2). Bonferroni post-hoc testing confirmed that these differences were driven by comparisons between 3a vs. 3b and 3a vs. 3c, with no significant difference between 3b and 3c. Both intra- and inter-observer agreement for IPN grading were robust: intra-observer agreement yielded a kappa value of 0.762 (95% CI: 0.714–0.809), and inter-observer agreement showed a kappa of 0.704 (95% CI: 0.653–0.755). Subgroup analysis demonstrated the highest consistency for 3a plaques (intra-observer κ = 0.784; inter-observer κ = 0.717), while 3c plaques exhibited slightly lower agreement (intra-observer κ = 0.686; inter-observer κ = 0.677).

### Risk factors predicting symptomatic plaques and the incremental value of IPN for reclassifying plaque-RADS categories

Of the 270 plaques, 80 (29.6%) were symptomatic. The distribution of symptomatic plaques across the original Plaque-RADS 3 subgroups was: 3a: 23/92 (25.0%), 3b: 46/146 (31.5%), 3c: 11/32 (34.4%). Multivariate logistic regression identified luminal stenosis (OR = 2.129, 95% CI: 1.429–3.172, *p* < 0.001), plaque thickness (OR = 2.429, 95% CI: 1.744–3.384, *p* < 0.001), plaque length (OR = 1.033, 95% CI: 1.018–1.049, *p* < 0.001), and IPN score (OR = 1.884, 95% CI: 1.152–3.081, *p* = 0.012) as independent predictors of symptomatic plaques (Table 3). The distribution of IPN grades across Plaque-RADS 3 subgroups revealed a clear gradient of symptomatic risk. Although the OR for IPN (1.884) may appear modest, it reflects the increased odds of symptomatic presentation per one-grade increase in the IPN score. For example, a plaque with IPN grade 3 has an estimated 6.69-fold higher odds of being symptomatic compared to a plaque with IPN grade 0 (1.884³).Following IPN-based reclassification of Plaque-RADS 3a plaques into 3a- (IPN 0–1) and 3a+ (IPN ≥ 2), the proportion of symptomatic plaques in the 3a- subgroup (18.9%, 7/37) was numerically lower than that in the 3a+ (29.1%, 16/55), 3b (31.5%, 46/146), and 3c (34.4%, 11/32) subgroups. Although chi-square testing indicated no statistically significant difference in symptomatic plaque rates across the four groups (*p* = 0.454), this statistically insignificance may result from limited sample size and should be interpreted cautiously. Combined with multivariate findings, it is believed that a substantial proportion of Plaque-RADS 3a plaques may be overestimated in terms of stroke risk (Table 4).

**Table 3:** Logistics regression analysis for predicting the symptomatic carotid plaques in the plaque 3 subgroup

	Odds ratio^*^	95% confidence interval		Wald	*p*-value
		Lower	Upper		
Stenosis	2.129	1.429	3.172	13.786	<0.001
Plaque-thickness	2.429	1.744	3.384	27.515	<0.001
Plaque-length	1.033	1.018	1.049	18.374	<0.001
IPN	1.884	1.152	3.081	6.372	0.012

**Table 4:** The proportion of symptomatic and asymptomatic plaques in different plaque-rads 3 groups according to the intraplaque neovascularization (IPN) grades

	3a-^*^	3a+^&^	3b-^*^	3b+^&^	3c-^*^	3c+^&^	Total
Symptomatic plaques	7 (18.9%)	16 (29.1%)	3 (27.3%)	43 (31.9%)	1 (50.0%)	10 (33.3%)	80
Asymptomatic Plaques	30 (81.1%)	39 (70.9%)	8 (72.7%)	92 (68.1%)	1 (50.0%)	20 (66.6%)	190
Cramer’s V value	0.115		0.026		0.085		0.287
P value	0.269		1.000		1.000		0.454

*: Plaques with intraplaque neovascularization grading of 0–1

&: Plaques with intraplaque neovascularization grading of 2–3

## Discussion

The Carotid Plaque-RADS system offers a standardized framework for assessing carotid plaque vulnerability and stroke risk. However, its clinical validation remains ongoing [7]. This study focused on the heterogeneous Plaque-RADS category 3 and demonstrated that CEUS-based IPN scoring can further stratify risk, particularly within the 3a subgroup.

Growing evidence highlights the prognostic significance of plaque vulnerability features—particularly intraplaque hemorrhage and thrombosis—in ischemic stroke risk prediction [16, 17]. IPN, a well-established marker of plaque instability, can be effectively visualized through CEUS, even showing the progression of vasa vasorum [18]. This modality demonstrates high concordance with histopathological microvascular density [12, 14, 19, 20]. Our multivariable analysis aligns with Zhang et al.‘s observation that higher IPN grades independently predict recent and future ischemic strokes in patients with mild carotid stenosis [21]. Similarly, Faggioli et al. reported greater CEUS enhancement intensity in symptomatic plaques, also correlating with thinner fibrous caps and pronounced inflammatory infiltration [22]. These findings are consistent with our major discoveries. Lyu et al. and Uchihara et al. further identified luminal-originating contrast entry patterns in plaques are strongly associated with plaque rupture and symptomatic presentations [23, 24]. This phenomenon cohered with our observation during the data collection of this study that most IPN 2–3 plaques exhibited adventitial shoulder enhancement. Notably, Camps-Renom et al. demonstrated IPN as an independent predictor of stroke recurrence (HR 6.57, 95% CI 1.66–26.01) after adjusting for stenosis severity [25], while Huang et al. recently emphasized Plaque-RADS’s superior prognostic value over conventional stenosis grading [26]. Collectively, both of the findings underscore the potential of integrating IPN assessment with Plaque-RADS for enhanced risk stratification.

Current Plaque-RADS 3 classification defined as “intermediate risk of stroke” may obscure clinically significant heterogeneity. For instance, 3a plaques with low IPN scores and intact fibrous caps may approximate low-risk profiles, whereas 3b/3c plaques with high IPN scores and thin/incomplete caps might align closer to high-risk categories. Additionally, although not specifically analyzed in this cohort, the presence of carotid web with superimposed plaque represented a totally different phenotype, which may confer a higher risk of stroke recurrence [27]. Dynamic change of IPN under pharmacologic treatments and its potential association with coronary artery disease and cardiovascular events also warrant consideration [28–30]. While current guidelines do not yet incorporate IPN-based stratification, our findings support the rationale for differential management. Plaques classified as 3a with low IPN (0–1) may represent a lower-risk subgroup suitable for extended surveillance intervals (e.g., 12–18 months) and conservative pharmacotherapy (e.g., statins alone). In contrast, 3a plaques with high IPN (≥ 2), as well as 3b and 3c plaques, may warrant intensified lipid-lowering or anti-inflammatory regimens, shorter follow-up intervals (e.g., 6 months), or referral for advanced imaging or surgical evaluation. This stratified approach could help reduce overtreatment while ensuring timely intervention for high-risk lesions.

This retrospective and single-center analysis carries inherent limitations, commonly including the selection bias and the compromised generalizability. The limited sample size—especially for 3c plaques (*n* = 32)—may hinder statistical power. Although interobserver agreement for IPN scoring was satisfactory (κ = 0.762), artificial intelligence-assisted quantification could further help [31]. The absence of multimodal imaging involvement (e.g., HR-MRI/CTA) restricts comprehensive plaque characterization [9, 32], and the 8-week window for defining symptomatic plaques precludes assessment of long-term IPN-stroke risk. Future studies could: (1) Integrate CEUS with multimodal imaging (e.g., MRI for IPH/LRNC/FC) to enable comprehensive plaque phenotyping and validate the incremental value of IPN within a multi-parametric framework; (2) Develop AI-driven IPN grading tools; (3) Conduct multi-center prospective trials evaluating IPN-stratified management on clinical endpoints (stroke incidence, revascularization rates).

## Conclusion

Our findings suggest that a substantial subset of Plaque-RADS 3a plaques (40.2%) with low IPN scores may represent a lower-risk phenotype, potentially meriting reclassification; however, longitudinal data linking IPN to future hard clinical outcomes (e.g., incident stroke or TIA) are crucial to establish a definitive causal inference and validate its prognostic utility.

## Data Availability

The dataset used for analysis during the current study are available from the corresponding author upon a reasonable request.
